# Occurrence, Sources, and Health Risks of Polycyclic Aromatic Hydrocarbons in Road Environments from Harbin, a Megacity of China

**DOI:** 10.3390/toxics11080695

**Published:** 2023-08-11

**Authors:** Jin-Nong Li, Ye Zhang, Jian-Xin Wang, Hang Xiao, Anatoly Nikolaev, Yi-Fan Li, Zi-Feng Zhang, Zhong-Hua Tang

**Affiliations:** 1 College of Chemistry, Chemical Engineering and Resource Utilization, Northeast Forestry University, Harbin 150040, China; 2021115383@nefu.edu.cn (J.-N.L.); yzhang@nefu.edu.cn (Y.Z.); wxx@nefu.edu.cn (J.-X.W.); 2 Key Laboratory of Plant Ecology, Northeast Forestry University, Harbin 150040, China; 3 Center for Excellence in Regional Atmospheric Environment, Institute of Urban Environment, Chinese Academy of Sciences, Xiamen 361021, China; hxiao@iue.ac.cn; 4 Institute of Natural Sciences, North-Eastern Federal University, 677000 Yakutsk, Russia; an.nikolaev@s-vfu.ru; 5 International Joint Research Center for Persistent Toxic Substances (IJRC-PTS), State Key Laboratory of Urban Water Resource and Environment, Harbin Institute of Technology, Harbin 150090, China; dr_li_yifan@163.com; 6 International Joint Research Center for Arctic Environment and Ecosystem (IJRC-AEE), Polar Academy, Harbin Institute of Technology, Harbin 150090, China; 7 Heilongjiang Provincial Key Laboratory of Polar Environment and Ecosystem (HPKL-PEE), Harbin Institute of Technology (HIT), Harbin 150090, China; 8 IJRC-PTS-NA, Toronto, ON M2N 6X9, Canada

**Keywords:** road dust, green belt soil, parking lot dust, PAHs, temporal and spatial variation, source apportionment, risk assessment

## Abstract

To obtain a comprehensive understanding about that occurrence, sources, and effects on human health of polycyclic aromatic hydrocarbons (PAHs) in road environmental samples from Harbin, concentrations of 32 PAHs in road dust, green belt soil, and parking lot dust samples were quantified. The total PAH concentrations ranged from 0.95 to 40.7 μg/g and 0.39 to 43.9 μg/g in road dust and green belt soil, respectively, and were dominated by high molecular weight PAHs (HMW-PAHs). Despite the content of PAHs in arterial roads being higher, the composition profile of PAHs was hardly influenced by road types. For parking lot dust, the range of total PAH concentrations was 0.81–190 μg/g, and three-ring to five-ring PAHs produced the maximum contribution. Compared with surface parking lots (mean: 6.12 μg/g), higher total PAH concentrations were detected in underground parking lots (mean: 33.1 μg/g). The diagnostic ratios of PAHs showed that petroleum, petroleum combustion, and biomass/coal combustion were major sources of PAHs in the samples. Furthermore, according to the Incremental Lifetime Cancer Risk model, the cancer risks of three kinds of samples for adults and children were above the threshold (10^−6^). Overall, this study demonstrated that PAHs in the road environment of Harbin have a certain health impact on local citizens.

## 1. Introduction

Polycyclic aromatic hydrocarbons (PAHs) are the ubiquitous persistent organic pollutants (POPs) and comprise two or more benzene rings. They are considered as the typical anthropogenic pollutants characterized by biological enrichment [1,2], hydrophobicity, anti-degradation [3,4], carcinogenicity, mutagenicity, and teratogenicity [5]. Long-term exposure to PAHs can cause damage to human organs including the skin, pulmonary, pancreas, etc. [6], and finally lead to multiple health problems, such as lung, breast, and bladder cancers [7]. Two- and three-ring PAHs are usually regarded as low molecular weight PAHs (LMW-PAHs), and that of four-, five-, and six-ring PAHs are high molecular weight PAHs (HMW-PAHs). LMW-PAHs are more water soluble and volatile, while HMW-PAHs have a tendency for deposition [8]. Among all PAHs, 16 PAHs were listed as priority pollutants by the United States Environmental Protection Agency [9,10], and BaA, BaP, BbF, BkF, CHR, DahA, and ICDP were classified as potential human carcinogens [11]. To identify the sources of PAHs is indispensable for the design of appropriate mitigative measures. In general, PAHs originated from multiple pathways sources of pollution, and mostly from human activities, such as vehicle emissions [12,13], the incomplete combustion of coal, woods, and fossil fuels, etc. [11]. PAHs produced from combustion processes are emitted into the air and descend to the land surfaces by dry and wet depositions, finally becoming street dust or being incorporated into soil [14]. One of the essential sources for PAH pollution in soil/road dust is atmospheric deposition [15].

Urban road dust is a complex mixture of motor vehicle exhaust, tire wear, soil, and airborne particles [16], and acts as a sink of substantial pollutants, which is an important medium for pollutant transport in urban areas [17]. A vast array of studies have reported the levels of PAHs in road dust from around the world [18,19,20,21]. And it is essential to mitigate the pollution of road dust by reducing pollutants such as PAHs in the various sources of road dust. For example, controlling the emissions of vehicle exhaust, such as exhaust gas from diesel engines, may not only improve engine durability but also reduce the content of pollutants [22]. Recent studies found that the addition of alcohols to diesel or biodiesel significantly reduced emissions of regulated pollutants and unregulated PAHs from diesel engine exhaust, which provides a positive measure to decrease PAH content in vehicle exhaust [23,24,25]. Parking lot dust seem to be a mixture of road dust from various sources, thus containing plenty of pollutants. Pedestrians, residents, and parking attendants were the potential groups that might be affected by parking lot dust [26]. In the megacities of China, numerous parking lots were built underground or in buildings, so were they in Harbin. The enclosed/semi-enclosed characteristic of these parking lots is not conducive to vehicle exhaust diffusion, resulting in an increase of vehicle-related pollutants in parking lot dust [27]. However, PAHs in parking lot dust has rarely gained the attention of researchers until now. Mahler et al. [28] indicated that the median concentration of total PAHs in dust from parking lots with a coal–tar-based sealcoat (CT) was much higher than from parking lots without CT; the presence of CT on a parking lot may influence the concentrations of PAHs in settled house dust from nearby apartments. In addition, the hydrophobicity and lipophilic nature of PAHs result in a tendency for PAHs to adhere to the organic matter in soil particles, which then contaminates soil [29]. Despite the fact that there are several reports on PAHs in agricultural soil, oasis soil, and coking plant soil [9], when it comes to green belt soil on roadsides, the related studies are very few. Therefore, it is necessary to investigate PAHs in green belt soils.

Distinct from previous studies, this is the first study containing a comparative analysis of PAHs in road dust, green belt soil, and parking lot dust; moreover, 32 PAHs (including 16 priority PAHs) are quantified by GC-MS/MS, which certainly might enrich the knowledge about PAHs in the environment. Therefore, the present study was aimed at assessing the occurrence of 32 PAHs in road environmental samples of Harbin (China) with the following objectives: (a) to assess the concentration distribution and composition profiles of PAHs in road dust, green belt soil, and parking lot dust; (b) to assess the effect of different road types and seasonal variations on the concentration of PAHs in road dust and green belt soil; (c) to calculate the concentration of carcinogenic PAHs in road environmental samples; (d) to identify the potential sources of PAHs in road environmental samples using diagnostic ratios; and (e) to evaluate the cancer risk of PAHs in road environmental samples from Harbin using an *ILCR* model.

## 2. Materials and Methods

### 2.1. Chemicals and Reagents

All standards for target analytes were purchased from AccuStandard Inc., New Haven, CT, USA, except for 1,2,3,4-Tetrahydronaphthalene (THN) an Retene (RET) (obtained from Dr. Ehrenstorfer Inc., Augsburg, Germany). Basic information and abbreviations of 32 PAHs are described in Table S1. The organic solvents used in this study were HPLC-grade, n-Hexane, Ethyl acetate, Dichloromethane, and Isooctane and were bought from Fisher Scientific (Fair Lawn, NJ, USA). Ultrapure water was prepared using a Milli-Q ultrapure system (Millipore; Billerica, MA, USA).

### 2.2. Study Area and Sample Collection

The study area was situated in Harbin city (44°04′ N–46°40′ N and 125°42′ E–130°10′ E), the capital of Heilongjiang Province, Northeast China [30]. In Harbin, the wintertime usually lasts for six months, from November to April. Harbin is a large city with a population of 3.8 million and with an annual mean temperature of 3.5 °C [31,32].

In total, 118 road dust and 82 green belt soil samples were collected in Harbin city during Autumn (October 2021) and Spring (May 2022). The sampling map and detailed information can be found in Supplementary Materials (Figure S1 and Table S2). Road dust (Rd) and green belt soil (Gbs) samples were collected at intervals of 20 m on each road according to 4 road types including arterial road (AR), sub-arterial road (SR), branch way (BW, no green belt soil), and highway (HW). In addition, parking lot dust samples (*n* = 45) were also collected from 5 surface parking lots (SP) and 10 underground parking lots (UP). An amount of 3–6 subsamples were mixed to obtain a single sample. Impurities such as defoliation, stone grains, and insect carcasses were removed from samples during the collection process. All samples were sealed in glass bottles and kept in a refrigerator at 4 °C. 

### 2.3. PAHs Extraction and Instrument Analysis

Then, 0.5 g of dust sample/1 g of soil sample was placed in a plastic centrifuge tube (15 mL) and extracted with 5 mL hexane/ethyl acetate/dichloromethane mixture (1:1:1, *v*/*v*/*v*) by shaking in a shaker (50/60 Hz) for 20 min, then transferred to a centrifuge and centrifuged for 5 min by 4000 r/min, and the supernatants were extracted in final. The extraction process was repeated three times. The supernatants were combined together and concentrated to 1 mL under a gentle stream of nitrogen, then were extracted with 2 mL of Isooctane and concentrated to 1 mL again, repeated twice. Finally, the extract was transferred to a brown bottle (1.5 mL) through a 0.22 nm filtration membrane of the organic system, and stored at −20 °C until analysis.

Analyses of the 32 polycyclic aromatic hydrocarbons (PAHs) were carried out using an Agilent 7890B GC system (Agilent Technologies, Santa Clara, CA, USA) interfaced with an Agilent 7000C electron ionization triple-quadrupole mass spectrometer (GC-EI-MS/MS; Agilent Technologies). The GC system was equipped with a DB-5 MS capillary column (30 m, 0.25 mm, inside diameter, 0.25 μm film thickness; J&W Scientific, Folsom, CA, USA). Specific parameters were as follows: (i) 1 μL of the aliquot was injected in pulsed splitless mode at 320 °C; (ii) the flow rate was 1.2 mL/min, (iii) the column heating procedure: increased the temperature from 80 °C to 220 °C at a rate of 20 °C/min, further increased to 240 °C at a rate of 5 °C/min, stayed at 240 °C for 3 min, finally increased to 300 °C at a rate of 40 °C/min, stayed at 300 °C for 18.5 min followed by a post-run time of 0.5 min at 300 °C. The retention time and collision energy for target compounds are listed in Table S3.

### 2.4. Quality Assurance and Quality Control (QA/QC)

All analyses and data were implemented with strict quality control. Two method blanks were processed for each batch of samples (*n* = 22). Mixed standards of 100PPB were used for quality control to check the consistency of data. The reported concentrations were corrected with blanks to eliminate the interferences and/or contaminations arising from the pre-treatment process. Quantifications of 32 PAHs were performed using a six-point calibration standard curve with concentrations ranging from 1 to 200 ng/mL (1, 5, 10, 50, 100, and 200 ng/mL). The limits of detection (LOD) and limits of quantification (LOQ) for individual PAHs can be found in Table S4.

### 2.5. Diagnostic Ratios for Source Apportionment

Diagnostic ratios of Ant/(Ant + Phe) vs. FlU/(FlU + PYR), BaA/(BaA + CHR) vs. FlU/(FlU + PYR), and ICDP/(ICDP + BahiP) vs. BaA/(BaA + CHR) were used as indications of PAH release to the environment in this study (anthracene (Ant), phenanthrene (Phe), fluoranthene (Flu), pyrene (PYR), benz[a]anthracene (BaA), chrysene (CHR), indeno [1,2,3-cd]pyrene (ICDP), benzo[g,h,i]perylene (BahiP)).

### 2.6. Health Risk Assessment

Toxic equivalent concentrations (*TEQ*) of 15 PAHs were calculated according to BaP equivalence method. The toxic equivalent factors (TEFs) used for the individual PAHs were as follows: acenaphthylene (Acy, 0.001), acenaphthene (Ace, 0.001), fluorene (Flo, 0.001), Phe (0.001), Ant (0.01), FLU (0.001), PYR (0.001), BaA (0.1), CHR (0.01), benzo[b]fluoranthene (BbF, 0.1), benzo[k]fluoranthene (BkF, 0.1), benzo[a]pyrene (BaP, 1), ICDP (0.1), and dibenz[a,h]anthracene (DahA, 1) and BahiP (0.01). The cumulative *TEQ* is given by Equation (1)

(1) $$TEQ=\sum (C_{i}\times TEF_{i})\;$$

where *C_i_* is the concentration of *i*th PAH and *TEF_i_* is the corresponding toxic equivalency factor.

The incremental lifetime cancer risk (*ILCR*) was calculated to assess the exposure risk of environmental PAHs. *ILCR*s for inhalation, ingestion, and dermal contact exposure to PAHs were quantified for each sample in Harbin city according to the following Equations (2)–(5):

(2) $$ILCR{\;}_{Ingestion}=CSF_{Ingestion}\times {\left(\frac{BW}{70}\right)}^{\frac{1}{3}}\times \frac{CS\times IR_{ingestion}\times EF\times ED}{BW\times AT\times 10^{6}}.$$

(3) $$ILCR{\;}_{Dermal}=CSF_{Dermal}\times {\left(\frac{BW}{70}\right)}^{\frac{1}{3}}\times \frac{CS\times SA\times AF\times ABS\times EF\times ED}{BW\times AT\times 10^{6}}\;$$

(4) $$ILCR{\;}_{Inhalation}=CSF_{Inhalation}\times {\left(\frac{BW}{70}\right)}^{\frac{1}{3}}\times \frac{CS\times IR_{inhalation}\times EF\times ED}{BW\times AT\times PEF}\;$$

(5) $$ILCR_{s}=\sum (ILCR_{ingestion}+ILCR_{dermal}+ILCR_{inhalation})\;$$

where CS is equal to *TEQ* calculated for each sample using the Equation (1), *CSF* is carcinogenic slope factor (mg/kg/day). The *CSF* was 7.3, 25, and 3.85 for *CSF_Ingestion_*, *CSF_Dermal_*, and *CSF_Inhalation_*, respectively. The meanings and detailed information of other exposure parameters are shown in Table S5.

### 2.7. Statistical Analysis

SPSS 27 and Origin 2023 were applied for statistical analysis of PAHs in road dust, green belt soil, and parking lot dust. All measured values below the LOQ were considered as zero for data analysis. Distribution of sampling sites was plotted using the ArcGIS 10.2 software.

## 3. Result and Discussion

### 3.1. Seasonal Variation and Spatial Distribution of PAHs

#### 3.1.1. In Road Dust and Green Belt Soil

In this study, 32 polycyclic aromatic hydrocarbons (PAHs, including 16 priority PAHs) were examined in road environment samples. The result showed that seven PAH species were not detected, including indene (Ind), naphthalene (NAP, one of the priority PAHs), 9,10-Diphenylanthracene (9,10-DPA), benzo[j]fluoranthene (BjF), dibenzo[a,l]pyrene (dBalP), dibenzo[a,i]pyrene (dBaiP), and dibenzo[a,h]pyrene (dBahP). Fluctuated concentrations of 25 PAHs were detected in almost all samples. The concentrations of 25 PAHs (including 15 priority PAHs) determined in road dust (*n* = 118), green belt soil (*n* = 82), and parking lot dust (*n* = 45) samples are summarized in Table 1. In general, the total PAHs mean concentration was higher in parking lot dust (24.1 μg/g) followed by green belt soil (9.88 μg/g) and road dust (8.73 μg/g). The distribution of PAHs with a different number of rings in the three kinds of road environmental samples revealed general uniformity with dominance of the four- and five-ring PAHs (Figure S2). RET was considered as a marker of biomass burning [31]. A recent study pointed out that RET was a possible cytotoxic compound since it caused DNA damage and cell death [33]. In this study, RET concentration was 216 ng/g in green belt soil, higher than that in road dust (166 ng/g, this study) and suburban surface soils (83.3 ng/g, Xi’an, China), but very much lower than that in road dust from Xi’an (2040 ng/g, similar to the result of parking lot dust in this study, which was 2013 ng/g) [34]. Moreover, 15 priority PAHs accounted for 76.6 ± 4.5%, 79.6 ± 5.9%, and 78.7 ± 9.0% of the total PAHs concentration in road dust, green belt soil, and parking lot dust, respectively, suggesting that priority PAHs might be the main pollutant contributing to PAHs contamination in the road environment. The concentration of priority PAHs in road dust was higher in Harbin (0.73–31.5 μg/g) than in Tianjin (China, 0.70–7.23 μg/g) [35], Kumasi (Ghana, 0.18–7.77 μg/g) [17], and Abadan (Iran, 0.40–11.8 μg/g) [36], whereas it was similar to that reported in Newcastle (UK, 0.59–46.0 μg/g) [16]. However, in terms of green belt soil, Pilková et al. [37] pointed out that the range of 16 PAH concentrations was 0.19–22.0 μg/g in roadside soils from Bratislava, Slovakia. Yu et al. [38] reviewed the pollution level of PAHs in urban soils of China and the concentrations of the total PAHs ranged from 0.065 to 23.6 μg/g in urban soil with a mean of 2.80 μg/g. In contrast, the concentration of priority PAHs was higher in this study, measuring from 0.31 to 35.3 μg/g (mean: 7.79 μg/g). This means that the green belt soils in Harbin were polluted by higher concentration of PAHs. The soil environment seems to be the ultimate sink as more than 90% of PAHs gets accumulated in the soil environment [9]. In parking lot dust, the mean value and range of total priority PAH concentrations were 11.341 μg/g and 0.68–150 μg/g, respectively. Higher content of PAHs in parking lot dust might be attributed to the vehicle driving conditions, such as the automobile decelerating and idling. An experimental study showed that the PAH concentrations were higher in deceleration and idle tests than in the uniform speed tests on gasoline and diesel vehicles [39].

**Figure 1:**
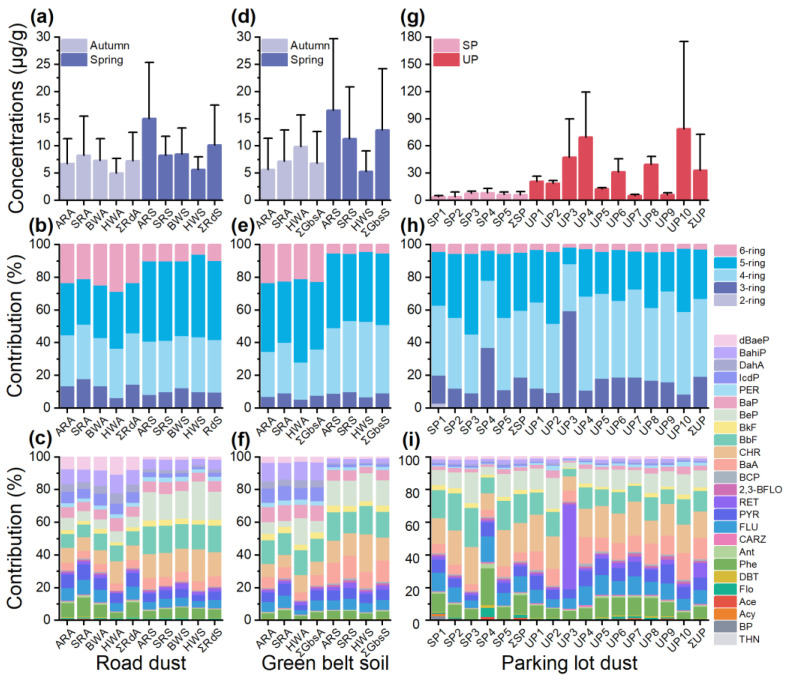
(**a**) The concentrations of total PAHs; (**b**) percentage composition of two-, three-, four-, five-, and six-ring PAHs; (**c**) percentage composition of individual PAHs in road dust (Rd) from Arterial road (AR), Sub-arterial road (SR), Branch way (BW), and Highway (HW) during autumn and spring; (**d**) the concentrations of total PAHs; (**e**) percentage composition of two-, three-, four-, five-, and six-ring PAHs; (**f**) percentage composition of individual PAHs in green belt soil (Gbs) from AR, SR, and HW during autumn and spring; (**g**) the concentrations of total PAHs; (**h**) percentage composition of two-, three-, four-, five-, and six-ring PAHs; (**i**) percentage composition of individual PAHs in surface parking lot (SP) and underground parking lot (UP) dust. ARA, SRA, BWA, and HWA: samples from different roads in autumn (ΣRdA/ΣGbsA represents all Rd/Gbs samples in autumn); ARS, SRS, BWS, and HWS: samples from different roads in spring (ΣRdS/ΣGbsS represents all Rd/Gbs samples in spring); ΣSP/ΣUP: all dust samples from SP/UP.

The concentration distribution, seasonal variation, and percentage composition of PAHs in road environmental samples collected within the main urban zone of Harbin, are presented in Figure 1. As for road dust (Figure 1a), even though the seasonal median values of the total PAH concentrations were similar: 7.78 μg/g in spring and 6.41 μg/g in autumn, the mean value obtained from the spring samples (10.20 μg/g) was almost 1.4 times higher than the mean value obtained from the autumn samples (7.26 μg/g). For green belt soil (Figure 1d), the mean content of the total PAHs was increased significantly in the spring season (12.90 μg/g) and was almost two times higher than that in the autumn season (6.86 μg/g). The median concentration of the total PAHs was higher in spring soil (8.92 μg/g) than in autumn soil (5.83 μg/g), which was different from the result in road dust. These results were consistent with previous reports on the seasonal variation of PAH concentrations in other cities. Gope et al. [40] reported total PAH concentrations that were nearly 2.1 times higher in winter than in summer due to the heating activities indoors and outdoors and the increase in volatile PAHs and high atmospheric stability, etc. Karaca et al. [41] also observed the higher concentration of PAHs in winter samples collected from Bursa, Turkey. Burning coal is the major method for house heating in the northern cities of China, and a large number of pollutants, including PAHs, are released during the combustion of coal [42], leading to increased PAH emissions in winter. Harbin (known as “Ice City”) has a six-month long heating period, which undoubtedly greatly increases the PAHs in the environment, which might be one of the important reasons for the higher concentration of PAHs in the road environment during spring. There was a study in Urumqi that showed that PAH concentrations decreased by 74% during the heating season, after the replacement of coal with natural gas for heating [43]. Thus, we urgently advocate for the use of clean energy for home heating to reduce PAH emissions in the heating season.

According to the number of benzene rings, 25 PAHs were classified into five groups as follow: two-ring PAHs, including THN and biphenyl (BP); three-ring PAHs, including Acy, Ace, Flo, dibenzothiophene (DBT), Phe, Ant, carbazole (CARZ), and RET; four-ring PAHs, including FLU, PYR, 2,3-Benzofluorene (2,3-BFLO), benzo[c]phenanthrene (BCP), BaA, and CHR; five-ring PAHs, including BbF, BkF, benzo[e]pyrene (BeP), BaP, perylene (PER), and DahA; six-ring PAHs, including ICDP, BahiP, and dibenzo[a,e]pyrene (dBaeP). There was a slightly different distribution of PAHs in autumn and spring; the percentages (<1%) of two-ring PAH concentrations were particularly low in both periods. As for road dust, the composition profile of the PAHs was dominated by four-ring (in order: CHR, PYR, FLU, BaA, etc.), five-ring (in order: BbF, BeP, BaP, etc.), and six-ring (in order: BahiP, dBaeP, ICDP) PAHs in autumn, making 85.2% of the total PAH level, followed by three-ring PAHs (accounting for 14.2%), and that in spring had the dominance of four-ring (in order: CHR, BAA, FLU, PYR, etc.) and five-ring (in order: BeP, BbF, BaP, etc.) PAHs, comprising 80.4% of the total PAH level; the percentage of three-ring PAHs concentration decreased to 9.5%, six-ring PAHs decreased from 23.1% in autumn to 9.71% in spring. A similar distribution of PAHs was observed in samples collected from green belt soil, with four- and five-ring PAHs being the dominant compounds, contributing to 69.7% and 85.6% of the total PAH concentrations in autumn and in spring, respectively, and six-ring PAHs decreased by 17.2% (22.3% in autumn). There was almost no variation for three-ring PAH concentrations. In addition, the major individual PAHs in autumn soil were BbF (0.97 μg/g), BahiP (0.75 μg/g), and BaP (0.64 μg/g), and those in spring were BbF (2.03 μg/g), BeP (2.00 μg/g), and CHR (1.83 μg/g). It should be noted that HMW PAHs had a relatively high tendency to accumulate in road dust and green belt soil, despite the fact that the six-ring PAHs slightly decreased in spring. A similar distribution of PAHs was determined in road dust samples of Abadan, Iran [36] and Northern Vietnam [44], which was >70% concentration of the total PAHs occupied by HMW-PAHs. The inherent properties of HMW-PAHs, such as resist transformation, degradation, translocation, and long-range transport [45], maybe contributed to the accumulation of PAHs in the road environment.

The samples collected from Arterial roads (AR), Sub-arterial roads (SR), Branch ways (BW), and Highways (HW) were used to elucidate the variation in PAHs concentrations for different types of roads. BW was set up for solving the traffic of localized areas, with the narrower road width and lower traffic flow, as a result that most BWs had no green belt soil. So, no green belt soil samples were collected from BW. As shown in Figure S3, the highest mean value of the total PAH concentrations was found in the AR samples with 10.9 μg/g in road dust and 11.1 μg/g in green belt soil, followed by SR, BW, and HW, respectively. For road dust, the mean concentration of the total PAHs in the AR samples was significantly higher than in other types of road samples (*p* < 0.001); this result was possibly attributable to a higher traffic load on ARs [46]. However, there was no significant difference between ARs and other roads for the total PAHs concentrations in green belt soil. Furthermore, the seasonal variation of the total PAHs concentrations in samples collected from the four kinds of roads showed inconsistencies (Figure 1a,d). The total PAH concentrations in different spring samples were in the following order: AR > SR > BW > HW, but the opposite trend was observed in green belt soil in autumn; meanwhile, the total PAH concentrations in road dust from ARs were lower than those from SRs and BWs in autumn. In addition, the type of roads might not have an impact on the composition profile of the PAHs (Figure 1b,c,e,f).

#### 3.1.2. In Parking Lot Dust

To evaluate the occurrence of PAHs in a parking lot, 32 PAHs (25 PAHs detected) in the dust of surface parking lots and underground parking lots were determined during the spring season. The total PAH concentrations in parking lot dust (mean: 24.1 μg/g) were higher than in road dust in spring (mean: 10.2 μg/g) and in green belt soil in spring (mean: 12.9 μg/g) (Figure 1g). The mean value of the total PAH concentrations was five times higher in underground parking lot dust (33.1 μg/g) than in surface parking lot dust (6.10 μg/g), which was caused by the higher concentrations of 3-, 4-, and 5-ring PAHs in underground parking lots (Figure S2). The enclosed environment of an underground parking lot might contribute to the accumulation of PAHs in this type of dust, and ultimately lead to a substantial increase in PAHs concentrations [27]. In this study, the mean content of PAHs for underground parking lots was slightly higher than the underground unloading area of the Forum Aveiro shopping centre (23.2 μg/g), and for the surface parking lot, it was much lower than the central parking facilities of the University of Aveiro campus (outdoor parking, 24.3 μg/g) [26]. The composition of PAHs in the two kinds of dust samples revealed general uniformity, with the sum of 3-, 4-, and 5-ring PAHs accounting for 94.5% and 97.0% of the total PAHs concentration in surface and underground parking lots, respectively (Figure 1h). The major individual PAHs were CHR, BbF, and BeP in both surface and underground parking lot dust (Figure 1i), with the range concentrations of the three PAHs in the surface parking lot being 0.14–2.26 μg/g (0.90 μg/g), 0.097–2.52 μg/g (0.85 μg/g), and 0.083–1.80 μg/g (0.51 μg/g), and in underground parking lot being 0.70–31.8 μg/g (3.92 μg/g), 0.31–30.8 μg/g (2.44 μg/g), and 0.31–24.2 μg/g (2.30 μg/g), respectively. To the best of our knowledge, currently, there are rarely reports about the concentration of PAHs in parking lot dust, and these results complement the relevant knowledge.

### 3.2. Abundance of Carcinogenic PAHs (CPAHs) and Toxic Equivalent Concentrations (TEQ)

**Figure 2:**
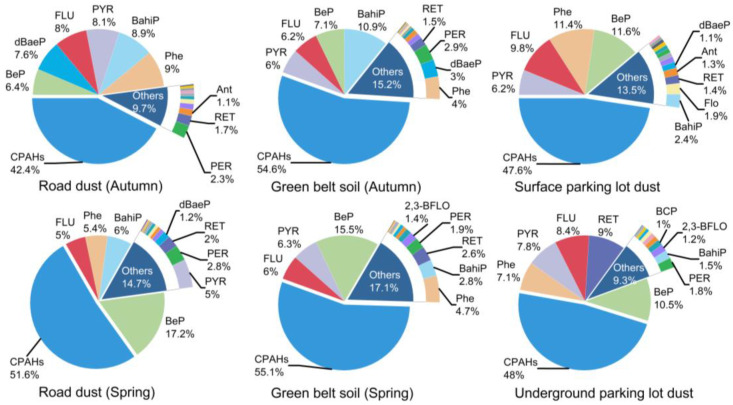
Abundance of carcinogenic PAHs (CPAHs) in road dust, green belt soil, and parking lot dust.

The abundance of carcinogenic PAHs (CPAHs) in road dust, green belt soil, and parking lot dust is graphically represented in Figure 2. The contribution of CPAHs in road dust in autumn and spring were 42.4% and 51.6%, respectively, and that in green belt soil in autumn and spring were 54.6% and 55.1%, respectively. The results showed that the proportion of CPAHs in road dust was higher during the spring compared with the autumn. Gope et al. [40] reported a seasonal variation in CPAH contents contrary to this study: the input of CPAHs was 45% lower in winter than in summer (53%) and during the monsoon season (47%) in the street dust of Durgapur, India. The contents of CPAHs contributed to 38% of the total PAHs in Tokyo, Japan [47]. It was interesting that despite the different conclusions obtained from another study conducted in India [11], there was no significant difference in CPAHs’ percentages during the summer (44%), the monsoon season (43%), and the winter (41%). The study by Pereira netto et al. might explain these differences; the content of the total PAHs and CPAHs were increased with the temperature reduction in road dust in Rio de Janeiro (a tropical city, which is less influenced by seasonal variation in emissions sources), Brazil. And even though the percentage of CPAHs varied from 29% to 45% of the total PAHs, it was not correlated with any of the selected temperatures, which might reflect the fact that CPAHs’ proportions were not affected by seasonal variations [48]. In addition, the abundance of CPAHs in surface parking lots was generally consistent with underground parking lot dust, which were 47.6% and 48.0%, respectively (Figure 2). In summary, CPAHs tend to accumulate in green belt soil rather than road dust and parking lot dust, in the road environment of Harbin.

The BaPeq concentrations of 15 priority PAHs were calculated to assess the PAHs toxicity in samples collected from road environments in Harbin. As shown in Table 1, the toxic equivalent concentrations (*TEQ*) of the 15 PAHs ranged between 0.13 μg/g and 4.89 μg/g in samples from road dust (mean: 1.07 μg/g), and between 0.019 μg/g and 5.88 μg/g in samples from green belt soil (mean: 1.26 μg/g), and between 0.051 μg/g and 19.1 μg/g in samples from parking lot dust (mean: 1.54 μg/g). Among all the samples, the highest value and the highest average value of the *TEQs* were both obtained from parking lot dust samples, suggesting that there was a high exposure risk of parking lot dust to human health. The average value of a *TEQ* was 1.07 μg/g in the road dust samples of Harbin, lower than that of Xi’an (1.15 μg/g), Guangzhou (2.76 μg/g), and Tianjin (4.55 μg/g) from China [17], and higher than that of Guwahati city (Brahmaputra Valley), which was found to be 0.34 ± 0.28 μg/g, 0.19 ± 0.16 μg/g, and 0.54 ± 0.50 μg/g in monsoon, post-monsoon, and pre-monsoon, respectively [49]. As for green belt soil, the average value of the *TEQ* in samples from Harbin (1.26 μg/g) was higher than that from Shanghai, China (0.89 μg/g) [50] and Lucknow, India (0.65 μg/g) [29]. In addition, seven CPAHs accounted for 99.0 ± 0.3%, 99.3 ± 0.3%, and 98.8 ± 0.7% of *TEQ*s, in road dust, green belt soil, and parking lot dust, respectively, and were therefore major *TEQ* contributors. Among the seven CPAHs, BaP is the most toxic PAH, characteristiced by its carcinogenicity and mutagenicity [51], DahA is predominantly produced by coal combustion [52] and had a TEF value of 1. The sum of BaP and DahA concentrations accounted for 12.2%, 11.6%, and 3.71% of 15 PAHs; however, the sum of the *TEQ* values of BaP and DahA accounted for 76.8%, 70.6%, and 49.8% of 15 PAHs, in road dust, green belt soil, and parking lot dust, respectively. Therefore, BaP and DahA were the main pollutants that caused a health risk. This phenomenon was also found in airborne fine particulate matter [53], wastewater treatment plant sludge, and sewer sediment [52]. As a result, it is necessary to control Bap and DahA in the environment.

### 3.3. Source Apportionment of PAHs

**Figure 3:**
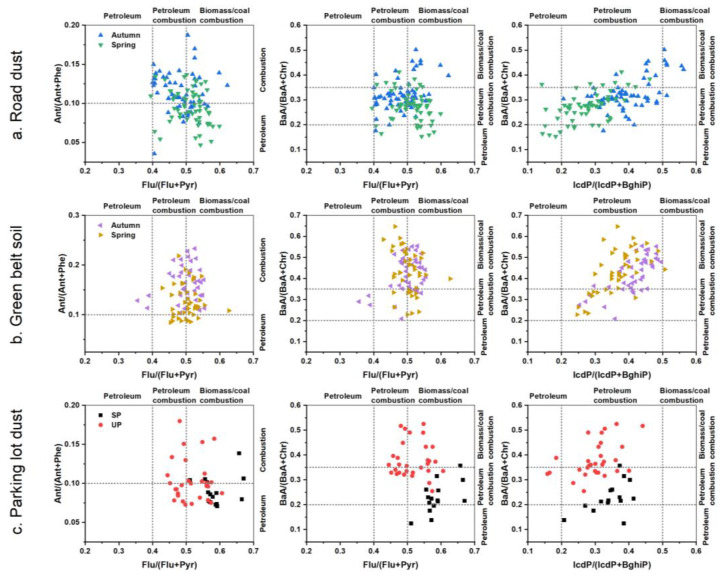
Diagnostic ratio of PAHs in (**a**) Road dust, (**b**) Green belt soil, and (**c**) Parking lot dust from Harbin, China. (SP: Surface parking lot, UP: Underground parking lot. The sample of green belt soil with Ant/(Ant + Phe) of 0.57 is not shown in the figure.).

The results of the diagnostic ratios (DRs) in samples from different road environments are presented in Table 2 and Figure 3. As for road dust, the results demonstrated that Ant/(Ant + Phe) ranged between 0.00 and 0.19 (mean: 0.10), Flu/(Flu + Pyr) ranged between 0.39 and 0.62 (mean, 0.50), BaA/(BaA + Chr) ranged between 0.15 and 0.50 (mean, 0.30), and (ICDP/ICDP + BahiP) ranged between 0.14 and 0.56. For green belt soil, the range of Ant/(Ant + Phe) was 0.08–0.57, with an average value of 0.15, that of Flu/(Flu + Pyr) was 0.36–0.63, with an average value of 0.50, that of BaA/(BaA + Chr) was 0.21–0.65, with an average value of 0.43, and that of (ICDP/ICDP + BahiP) was 0.25–0.51, with an average value of 0.39. For parking lot dust, the range of these ratios were 0.07–0.18 (mean: 0.10), 0.44–0.67 (mean: 0.54), 0.12–0.53 (mean: 0.33), and 0.16–0.44 (mean: 0.32) for Ant/(Ant + Phe), Flu/(Flu + Pyr), BaA/(BaA + Chr), and (ICDP/ICDP + BahiP), respectively. These results suggested that there were three diagnostic ratios in road dust, green belt soil, and parking lot dust that were similar, i.e., Ant/(Ant + Phe), Flu/(Flu + Pyr), and (ICDP/ICDP + BahiP) (Figure 3). The values of the three ratios in most of samples were concentrated at 0.1 (<0.1 was petroleum, >0.1 was combustion), 0.5 (0.4–0.5 was petroleum combustion, >0.5 was biomass/coal combustion), and 0.2–0.5 (petroleum combustion) for Ant/(Ant + Phe), Flu/(Flu + Pyr), and (ICDP/ICDP + BahiP), respectively. The different ratio results of BaA/(BaA + Chr) were observed in the three road environmental samples, the values of BaA/(BaA + Chr) were concentrated to 0.2–0.35 (petroleum combustion), >0.35 (biomass/coal combustion), and >0.2 (petroleum combustion and biomass/coal combustion) in road dust, green belt soil, and parking lot dust, respectively. After further evaluation (Figure 3), the results of Flu/(Flu + Pyr) vs. BaA/(BaA + Chr) indicated that petroleum combustion and biomass/coal combustion were the main sources of PAHs, and the same main sources of PAHs were observed from the results of IcdP/(IcdP + BahiP) vs. BaA/(BaA + Chr). However, the sources for Flu/(Flu + Pyr) vs. Ant/(Ant + Phe) were mainly related to petroleum, petroleum combustion, and biomass/coal combustion. In conclusion, petroleum, petroleum combustion, and biomass/coal combustion were the main sources of PAHs in the urban road environment samples from Harbin. Wu et al. [35] reported that PAHs in urban road dust from Tianjin also mainly originated from a mixture of petroleum, petroleum combustion, and biomass/coal combustion sources. However, it was worthily noted that only the results of the Ant/(Ant + Phe) pointed out petroleum sources as the main sources for PAHs. The ratio of Ant/(Ant + Phe) might be a rather inaccurate source identification tool due to the unstable ratio of low molecular mass compounds (more susceptible to the photodegradation), and might obtain the wrong results [54]. On the other hand, PAHs usually occur as a mixture, the compositions of multiple sources of PAHs overlap [47], and the emission and degradation of an individual PAH may produce substantial variability with the distance from emission sources, various combustion conditions, post-emission degradation, and meteorological conditions [55]. These limitations could potentially intensify the unreliability of DRs.

### 3.4. Carcinogenic Risk Assessments of PAHs in Road Dust, Green Belt Soil and Parking Lot Dust

In this study, the incremental lifetime cancer risk (*ILCR*) model was adopted to assess the human health risks of that via ingestion, inhalation, and dermal exposure. The *ILCR* values of children and adults in samples from road dust, green belt soil, and parking lot dust are given in Table 3. The average values of the total *ILCR*s for adults were 5.69 × 10^−6^, 6.72 × 10^−6^, and 8.2 × 10^−6^, and for children, they were 5.89 × 10^−6^, 6.96 × 10^−6^, and 8.50 × 10^−6^ in road dust, green belt soil, and parking lot dust, respectively, and all exceeded the threshold of 10^−6^. The highest value of total cancer risk in all the samples from a road environment was found to be 1.02 × 10^−4^ for adults and 1.06 × 10^−4^ for children, produced in the underground parking lot samples. Li et al. [46] investigated the cancer risk of 16 PAHs in 212 road dust samples collected from 53 cities across China; the results indicated that most cities (80%) from China were at a moderate potential carcinogenic risk, which was consistent with this study. Additionally, the mean cancer risks through inhalation were approximately 10^−10^ and 10^−11^ for adults and children, both about 4–5 orders of magnitude less than ingestion and dermal contact pathways (Table 3). The route of dermal contact resulted in the highest cancer risk, which accounted for 64% and 56% of the total risk for adults and children, whereas ingestion (36% for adults, 44% for children) was a relatively minor contributor of potential cancer risk. Therefore, ingestion and dermal contact were the main routes of exposure to PAHs [56]. In general, the health risk assessment suggested that PAHs in road environmental samples produced potential cancer risks for urban residents living in Harbin; meanwhile, for parking lot dust, the cancer risk was highest and the existence of samples with high cancer risk may produce a serious threat to people living around a parking lot.

**Figure 4:**
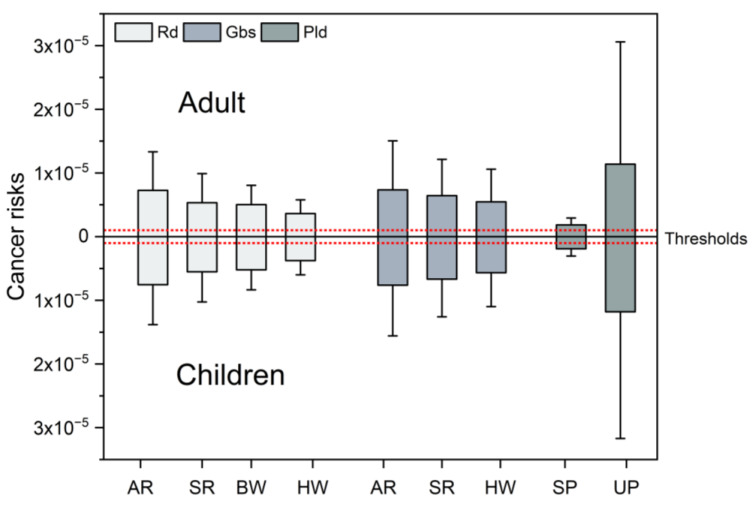
The incremental lifetime cancer risk (*ILCR*) estimated for children and adult populations.

The variations in *ILCR*s with the road types are graphically represented in Figure 4. There existed a negligible discrepancy in the probability of suffering from cancer via contact to the road samples from Harbin between adults and children. As for both road dust and green belt soil, the order of the cancer risk of the samples from different roads was AR (Rd: 7.27/7.53 × 10^−6^ for adults/children, Gbs: 7.35/7.62 × 10^−6^ for adults/children) > SR (Rd: 5.33/5.53 × 10^−6^, Gbs: 6.44/6.67 × 10^−6^) > BW (Rd: 5.03/5.21 × 10^−6^, no green belt soil) > HW (Rd: 3.62/3.75 × 10^−6^, Gbs: 5.46/5.65 × 10^−6^), and was consistent with the variations in total PAH concentrations on corresponding roads. The mean total *ILCR* values of PAHs in four kinds of samples divided by road class all got ahead of 10^−6^. Furthermore, for both road dust and green belt soil, the risk of carcinogenesis was less different among the autumn and spring (Figure S4). As for parking lot dust, underground parking lots had higher health risks compared with surface parking lots (Figure 4), which was also consistent with the results of concentrations.

## 4. Conclusions

The concentrations of PAHs in the samples indicated that the road environment of Harbin was suffering from the contamination of PAHs. The total PAH concentrations of different samples had the following order: road dust < green belt soil < parking lot dust. For road dust and green belt soil from Harbin, the total PAHs concentrations were influenced by road types, however the compositional features of PAHs were not influenced by road types. Higher PAH concentrations were observed in the spring samples. As for parking lot dust, the total PAH concentrations were higher in the underground parking lot rather than the surface parking lot, at a rate of five times higher. CHR, BbF, and BeP were dominant in both surface and underground parking lot dust. The abundance of carcinogenic PAHs showed that the toxic potency of the samples in the green belt soil was higher than in the road dust and the parking lot dust with an insignificant seasonal variation, and CPAHs had the tendency to accumulate in soil for the road environment of Harbin. The results of diagnostic ratios suggested that the mixture source of petroleum, petroleum combustion, and coal/biomass combustion was the main source for PAHs in the road environmental samples of Harbin. The *ILCR* model indicated that a higher carcinogenic risk for children as well as adults was found in parking lot dust, especially in the underground parking lot.

In sum, the underground parking lot dust samples had the highest total PAH concentrations; PAHs in road dust, green belt soil, and parking lot dust all have a potential cancer risk, which might have a certain impact on resident health. Furthermore, in order to clarify whether the high concentrations of PAHs in underground parking lot dust are widespread, we propose to monitor PAHs in underground parking lot dust from other countries in subsequent studies.

## Figures and Tables

**Table 1:** Statistical description of PAH concentrations in road dust, green belt soil, and parking lot dust.

Compounds (ng/g)	Road Dust			Green Belt Soil			Parking Lot Dust		
	Range	Median	Mean	Range	Median	Mean	Range	Median	Mean
THN	ND-69.5	3.80	6.89	ND-35.7	1.51	3.8	0.87–38.6	10.2	10.8
BP	ND-251	27.2	38.6	1.20–229	11.5	24.8	9.85–328	49.4	77.4
Acy	3.66–139	24.4	30	1.83–93.4	21.5	28.7	8.94–266	33.4	56.5
Ace	ND-79.3	14.4	17.6	0.51–52.7	9.36	13.2	4.08–337	20.8	35.6
Flo	ND-217	45.0	57.3	1.54–198	22.2	35.9	7.00–1116	113	186
DBT	ND-89.9	21.7	26.9	0.01–69.4	12.2	17.4	3.62–492	69.1	114
Phe	ND-4117	470	607	22.3–2206	305	442	93.0–7104	1224	1788
Ant	ND-441	48.3	70.7	3.64–401	51.7	71.2	10.8–1358	124	219
CARZ	5.20–144	19.6	28.5	1.41–116	14.6	20.6	2.68–1125	60.9	169
FLU	53.6–3441	446	546	25.0–3235	455	601	93.0–12,489	1120	2059
PYR	51.2–3113	420	547	29.0–3744	435	614	89.0–12,680	1103	1836
RET	0.33–1276	104	166	4.05–2080	92	216	14.7–62,859	223	2013
2,3-BFLO	6.95–597	54.5	74.6	3.95–956	75.1	118	4.94–2724	135	290
BCP	2.98–302	34.3	47.3	2.25–605	48.2	83	4.41–1908	114	238
BaA	44.3–3880	341	486	19.7–8833	628	1056	19.8–34,050	1101	2949
CHR	114–5525	860	1076	55.1–5815	739	1193	139–31,787	2329	3989
BbF	99.4–6388	758	1150	17.3–13,760	1007	1496	97.0–30,776	1809	3117
BkF	24.7–1558	183	266	7.87–2969	234	375	23.2–5631	259	510
Bep	54.7–6654	688	1110	26.2–6281	665	1241	83.6–24,179	1379	2543
BaP	57.9–2819	399	552	11.6–3432	444	720	26.6–10,811	269	713
PER	22.0–1330	158	224	6.63–1128	167	221	8.08–6564	128	403
ICDP	33.5–2619	220	367	8.42–2231	199	393	22.5–1459	98.9	178
DahA	24.5–1327	195	266	ND-1111	91.6	186	3.47–724	57.0	94.7
BahiP	103–2567	455	629	11.6–2503	364	557	36.0–1851	229	370
dBaeP	30.3–3665	180	336	6.64–957	86.2	142	0.79–530	91.8	134
ΣLMW	15.8–5549	798	1043	42.2–4414	560	874	155–67,099	2084	4672
ΣHMW	935–37,204	5787	7683	300–41,622	6329	9003	654–177,409	9357	19,432
ΣCPAHs	466–21,637	3021	4166	131–28,936	3927	5421	334–115,243	6094	11,554
Σ15 PAHs	734–31,547	5179	6667	314–35,291	5770	7788	677–149,876	11,341	18,108
ΣPAHs	950–40,749	6824	8726	394–43,900	7094	9878	810–190,222	13,627	24,105
*TEQ*	134–4892	773	1065	18.6–5883	851	1258	51.0–19,109	659	1536

15 PAHs: 15 priority PAHs (Acy, Ace, Flo, Phe, Ant, FLU, PYR, BaA, CHR, BbF, BkF, BaP, ICDP, DahA, BahiP). CPAHs: Concentrations of seven carcinogenic PAHs. LMW: Concentrations of low molecular weight PAHs. HMW: Concentrations of high molecular weight PAHs. *TEQ*: A sum of toxic equivalent quantity of 15 PAHs (Acy, Ace, Flo, Phe, Ant, FLU, PYR, BaA, CHR, BbF, BkF, BaP, ICDP, DahA, BahiP). ND: Not detected.

**Table 2:** Diagnostic ratios of PAHs.

Ratios	Road Dust				Green Belt Soil				Parking Lot Dust			
	Min	Max	Median	Mean	Min	Max	Median	Mean	Min	Max	Median	Mean
Ant/(Ant + Phe)	0.00	0.19	0.11	0.10	0.08	0.57	0.14	0.15	0.07	0.18	0.10	0.10
Flu/(Flu + Pyr)	0.39	0.62	0.50	0.50	0.36	0.63	0.50	0.50	0.44	0.67	0.56	0.54
BaA/(BaA + Chr)	0.15	0.50	0.30	0.30	0.21	0.65	0.43	0.43	0.12	0.53	0.34	0.33
ICDP/(ICDP + BahiP)	0.14	0.56	0.34	0.34	0.25	0.51	0.40	0.39	0.16	0.44	0.32	0.32

**Table 3:** The incremental lifetime cancer risk (*ILCR*) of PAHs in road dust, green belt soil, and parking lot dust.

*ILCRs*		Road Dust		Green Belt Soil		Parking Lot Dust	
		Adult	Children	Adult	Children	Adult	Children
Ingestion	Min	2.59 × 10^−7^	3.32 × 10^−7^	3.59 × 10^−8^	4.6 × 10^−8^	9.81 × 10^−8^	1.26 × 10^−7^
	Max	9.4 × 10^−6^	1.2 × 10^−5^	1.13 × 10^−5^	1.45 × 10^−5^	3.67 × 10^−5^	4.7 × 10^−5^
	Mean	2.05 × 10^−6^	2.62 × 10^−6^	2.42 × 10^−6^	3.1 × 10^−6^	2.95 × 10^−6^	3.78 × 10^−6^
	Median	1.49 × 10^−6^	1.9 × 10^−6^	1.64 × 10^−6^	2.1 × 10^−6^	1.27 × 10^−6^	1.62 × 10^−6^
Dermal	Min	4.61 × 10^−7^	4.14 × 10^−7^	6.38 × 10^−8^	5.73 × 10^−8^	1.74 × 10^−7^	1.57 × 10^−7^
	Max	1.67 × 10^−5^	1.5 × 10^−5^	2.01 × 10^−5^	1.81 × 10^−5^	6.53 × 10^−5^	5.86 × 10^−5^
	Mean	3.64 × 10^−6^	3.27 × 10^−6^	4.3 × 10^−6^	3.86 × 10^−6^	5.25 × 10^−6^	4.71 × 10^−6^
	Median	2.64 × 10^−6^	2.37 × 10^−6^	2.91 × 10^−6^	2.61 × 10^−6^	2.25 × 10^−6^	2.02 × 10^−6^
Inhalation	Min	2.01 × 10^−11^	6.44 × 10^−12^	2.78 × 10^−12^	8.91 × 10^−13^	7.61 × 10^−12^	2.44 × 10^−12^
	Max	7.29 × 10^−10^	2.34 × 10^−10^	8.77 × 10^−10^	2.81 × 10^−10^	2.85 × 10^−9^	9.12 × 10^−10^
	Mean	1.59 × 10^−10^	5.09 × 10^−11^	1.88 × 10^−10^	6.01 × 10^−11^	2.29 × 10^−10^	7.33 × 10^−11^
	Median	1.15 × 10^−10^	3.69 × 10^−11^	1.27 × 10^−10^	4.07 × 10^−11^	9.83 × 10^−11^	3.15 × 10^−11^
Cancer risk	Min	7.20 × 10^−7^	7.46 × 10^−7^	9.97 × 10^−8^	1.03 × 10^−7^	2.72 × 10^−7^	2.82 × 10^−7^
	Max	2.61 × 10^−5^	2.71 × 10^−5^	3.14 × 10^−5^	3.25 × 10^−5^	1.02 × 10^−4^	1.06 × 10^−4^
	Mean	5.69 × 10^−6^	5.89 × 10^−6^	6.72 × 10^−6^	6.96 × 10^−6^	8.2 × 10^−6^	8.5 × 10^−6^
	Median	4.13 × 10^−6^	4.28 × 10^−6^	4.55 × 10^−6^	4.71 × 10^−6^	3.52 × 10^−6^	3.65 × 10^−6^

## Data Availability

The data collected are the property of our research center but will be made available by the corresponding author when requested.

## Supplementary Materials

The following supporting information can be downloaded at: https://www.mdpi.com/article/10.3390/toxics11080695/s1, Figure S1: Sampling sites of road dust, green belt soil, and parking lot dust in Harbin, Northeast China; Figure S2: The concentrations of PAHs with a different number of aromatic rings in road dust, green belt soil, and parking lot dust; Figure S3: The concentrations of PAHs in AR, SR, BW, and HW during autumn and spring; Figure S4: Seasonal variations of carcinogenic risk values; Table S1: Basical information for 32 PAHs; Table S2: Detailed information of sampling sites; Table S3: The optimized GC-MS/MS parameters for PAHs; Table S4: Limits of Detection (LOD) and Limits of Quantification (LOQ) of individual PAHs in dust and soil samples; Table S5: The values of exposed parameters used for the incremental lifetime cancer risk assessment (ILCR). Re [35].
