# Supplementary material for: Occurrence, Sources, and Health Risks of Polycyclic Aromatic Hydrocarbons in Road Environments from Harbin, a Megacity of China
Source: Toxics. 2023 Aug 11;11(8):695. doi: 10.3390/toxics11080695 (PMC10458957; doi:10.3390/toxics11080695)
Supplement: Supplementary file 1 [file toxics-11-00695-s001.zip › toxics-2507101-supplementary.pdf]

toxics

MDPI

Supplementary materials

Occurrence, Sources, and Health Risks of Polycyclic Aromatic Hydrocarbons in Road Environments from Harbin, a Megacity of China

Jinnong Li, Ye Zhang, Jianxin Wang, Hang Xiao, Anatoly Nikolaev, Yi-Fan Li, Zi-Feng Zhang and Zhong-Hua Tang

Table S1. Basical information for 32 PAHs

PAHs	Abbr	Molecular formula	Molecular weight	Structure	Log K_{ow}[a]	Water solubility (mg/L)
1,2,3,4-Tetrahydronaphthalene	THN	$C_{10}H_{12}$	132.202	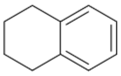	3.49	94.26
Indene	Ind	C_9H_8	116.160	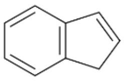	3.25	332.4
Naphthalene	NAP	$C_{10}H_8$	128.171	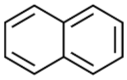	3.17	142.1
Biphenyl	BP	$C_{12}H_{10}$	154.208	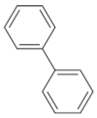	3.76	29.01
Acenaphthylene	Acy	$C_{12}H_8$	152.192	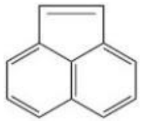	3.94	2.487
Acenaphthene	Ace	$C_{12}H_{10}$	154.208	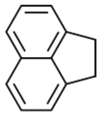	3.92	2.534
Fluorene	Flo	$C_{13}H_{10}$	166.219	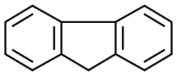	4.02	1.339
Dibenzothiophene	DBT	$C_{12}H_8S$	184.257	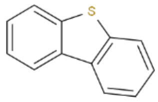	4.29	0.883
Phenanthrene	Phe	$C_{14}H_{10}$	178.229	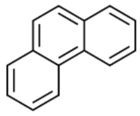	4.35	0.677
Anthracene	Ant	$C_{14}H_{10}$	178.229	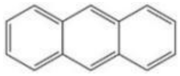	4.35	0.959
Carbazole	CARZ	$C_{12}H_9N$	167.207	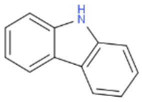	3.29	7.607
Fluoranthene	FLU	$C_{16}H_{10}$	202.251	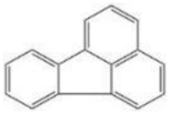	4.93	0.130

Pyrene	PYR	$C_{16}H_{10}$	202.251	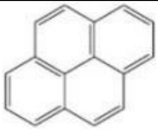	4.93	0.225
Retene	RET	$C_{18}H_{18}$	234.335	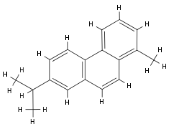	6.35	0.009
2,3-Benzofluorene	2,3-BFLO	$C_{17}H_{12}$	216.277	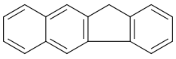	5.19	0.033
Benzo[c]phenanthrene	BCP	$C_{18}H_{12}$	228.288	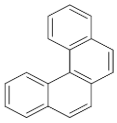	5.52	0.026
Benz[a]anthracene	BaA	$C_{18}H_{12}$	228.288	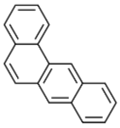	5.52	0.029
Chrysene	CHR	$C_{18}H_{12}$	228.288	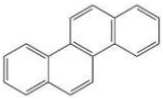	5.52	0.026
Benzo[j]fluoranthene	BjF	$C_{20}H_{12}$	252.309	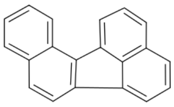	5.78	0.021
Benzo[b]fluoranthene	BbF	$C_{20}H_{12}$	252.309	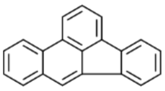	6.11	0.021
Benzo[k]fluoranthene	BkF	$C_{20}H_{12}$	252.309	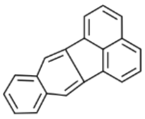	6.11	0.011
Benzo[e]pyrene	BeP	$C_{20}H_{12}$	252.309	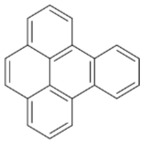	6.11	0.006
Benzo[a]pyrene	BaP	$C_{20}H_{12}$	252.309	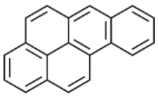	5.99	0.174
Perylene	PER	$C_{20}H_{12}$	252.309	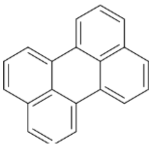	6.11	0.225
9,10-Diphenylanthracene	9,10-DPA	$C_{26}H_{18}$	330.421	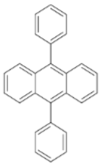	7.87	3.756 e-005

Indeno[1,2,3-cd]pyrene	ICDP	$C_{22}H_{12}$	276.331	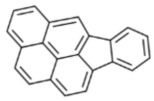	6.76	0.028
Dibenz[a,h]anthracene	DahA	$C_{22}H_{14}$	278.347	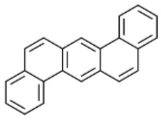	6.70	0.003
Benzo[g,h,i]perylene	BahiP	$C_{22}H_{12}$	276.331	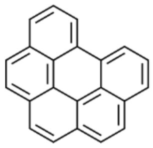	6.70	0.002
Dibenzo[a,l]pyrene	dBalP	$C_{24}H_{14}$	302.368	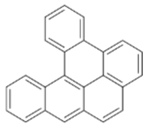	7.28	2.404 e-004
Dibenzo[a,e]pyrene	dBaeP	$C_{24}H_{14}$	302.368	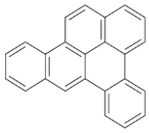	7.28	2.404 e-004
Dibenzo[a,i]pyrene	dBaiP	$C_{24}H_{14}$	302.368	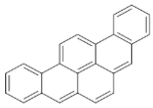	7.28	5.544 e-004
Dibenzo[a,h]pyrene	dBahP	$C_{24}H_{14}$	302.368	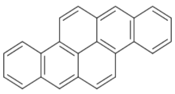	7.28	5.544 e-004

Note: *K*ow = Octanol-water Partition coefficient; a: predicted using EPI suite V4.1

Table S2. Detailed information of sampling sites

**Samples sites**	**Road designations**					
Arterial road (AR, n=6)	Youyixi road	Zhongshan road	Hexing road	Hongqi street	Xianfeng road	Jichang road
Sub-arterial road (SR, n=6)	Xinyang road	Lijiang road	Haping road	Songhai road	Hadong road	Nanzhi road
Branch way (BW, n=6)	Guxin road	Herun street	Xusheng street	Xuefudong road	Xilong street	Daokou street
Highway (HW, n=1)	Airport expressway					
Surface parking lot (n=5)	Hongbo	Maidelong	Wangfujing	Meikailong	Zhongyangdajie	
Underground parking lot (n=10)	Leso	Guxiang	Sifangtai	Yuanda	Baisheng	
	Maikaile	Kaide	Darunfa	Yijiajiaju	Jinjuewanxing	

Table S3. The optimized GC-MS/MS parameters for PAHs.

PAHs	Retention time (min)	Quantifier Transition (m/z)	Collision energy (eV)	Qualifier Transition (m/z)	Collision energy (eV)
1,2,3,4-Tetrahydronaphthalene	3.529	132→104	15	104→78	10
Indene	3.532	115→89	20	115→65	25
Naphthalene	3.932	128→102	20	128→127	20
Biphenyl	5.232	154→153	15	154→152	30
Acenaphthylene-D8	5.850	160→158	25	160→132	30
Acenaphthylene	5.890	152→150	25	152→151	25
Acenaphthene-D10	5.926	162→160	30	162→158	30
Acenaphthene	5.996	153→152	25	153→151	25
Fluorene-D10	6.580	176→174	20	176→172	40
Fluorene	6.660	166→165	25	165→163	30
Dibenzothiophene	7.816	184→152	35	184→139	40
Phenanthrene-D10	7.904	188→160	30	188→184	40
Phenanthrene	8.024	178→176	25	178→152	25
Anthracene	8.091	178→176	25	178→152	25
Carbazole	9.292	167→166	20	167→139	35
Fluoranthene-D10	9.402	212→208	40	212→210	30
Fluoranthene	10.402	202→200	35	202→201	25
Pyrene-D10	10.845	212→208	40	212→210	40
Pyrene	10.925	202→200	35	202→201	25
Retene	11.429	219→204	15	234→219	15
2,3-Benzofluorene	12.077	216→215	25	215→213	40
Benzo(c)phenanthrene	14.390	228→227	20	228→226	45
Benz(a)anthracene-D12	15.011	240→236	40	240→212	40
Benz(a)anthracene	15.091	228→226	30	228→202	30
Chrysene-D12	15.061	240→236	40	240→212	30
Chrysene	15.161	228→226	30	228→202	30
Benzo[b]Fluoranthene-D12	17.163	264→260	40	264→236	40
Benzo[k]Fluoranthene-D12	17.218	264→260	40	264→236	40
Benzo(j)fluoranthene	17.241	252→250	40	250→248	45
Benzo[b]fluoranthene	17.243	252→250	30	252→226	25
Benzo[k]fluoranthene	17.288	252→250	30	252→226	25
Benzo(e)pyrene	17.829	252→250	45	250→248	45
Benzop[a]pyrene-D12	17.880	264→260	40	264→236	40
Benzo[a]pyrene	17.960	252→250	30	252→226	25
Perylene-D	18.129	264→260	30	264→262	30
Perylene	18.169	252→250	45	250→248	45
9,10-Diphenylanthracene	18.359	252→250	40	330→252	35
Indeno[1,2,3-cd]pyrene-D12	21.280	288→284	40	288→286	40

Dibenzo[a,h]anthracene-D14	21.334	292→288	40	292→290	30
Indeno[1,2,3-cd]pyrene	21.360	276→274	45	276→272	50
Dibenz(a,h)anthracene	21.434	278→274	55	278→276	50
Benzo[g,h,i]perylene-D12	22.164	288→284	40	288→286	30
Benzo[g,h,i]perylene	22.254	276→274	45	276→272	50
Dibenzo(a,l)pyrene	27.591	302→300	45	302→301	20
Dibenzo(a,e)pyrene	31.066	302→300	45	300→298	40
Dibenzo(a,i)pyrene	32.170	302→300	50	300→298	45
Dibenzo(a,h)pyrene	32.749	302→300	40	300→298	40

Table S4. Limits of Detection (LOD) and Limits of Quantification (LOQ) of individual PAHs in dust and soil samples.

**PAHs**	**Dust**		**soil**	
	**LOD (ng/g)**	**LOQ (ng/g)**	**LOD (ng/g)**	**LOQ (ng/g)**
1,2,3,4-Tetrahydronaphthalene	0.50	1.66	0.25	0.83
Indene	2.98	9.92	1.49	4.96
Naphthalene	0.01	0.04	0.01	0.02
Biphenyl	0.64	2.14	0.32	1.07
Acenaphthylene	0.11	0.35	0.05	0.18
Acenaphthene	0.46	1.52	0.23	0.76
Fluorene	0.10	0.32	0.05	0.16
Dibenzothiophene	0.01	0.04	0.01	0.02
Phenanthrene	0.03	0.10	0.01	0.05
Anthracene	0.03	0.10	0.02	0.05
Carbazole	0.04	0.14	0.02	0.07
Fluoranthene	0.01	0.03	0.01	0.02
Pyrene	0.01	0.03	0.004	0.01
Retene	0.03	0.10	0.01	0.05
2,3-Benzofluorene	0.78	2.59	0.39	1.30
Benzo(c)phenanthrene	0.02	0.05	0.01	0.03
Benz(a)anthracene	0.05	0.16	0.02	0.08
Chrysene	0.05	0.16	0.02	0.08
Benzo(j)fluoranthene	0.048	0.16	0.02	0.08
Benzo[b]fluoranthene	0.06	0.19	0.03	0.09
Benzo[k]fluoranthene	0.05	0.18	0.03	0.09
Benzo(e)pyrene	0.11	0.36	0.05	0.18
Benzo[a]pyrene	0.79	2.64	0.40	1.32
Perylene	0.23	0.76	0.11	0.38
9,10-Diphenylanthracene	0.53	1.76	0.26	0.88
Indeno[1,2,3-cd]pyrene	0.14	0.48	0.07	0.24
Dibenz(a,h)anthracene	0.03	0.10	0.02	0.05
Benzo[g,h,i]perylene	0.03	0.10	0.01	0.05

Dibenzo(a,l)pyrene	0.03	0.11	0.02	0.06
Dibenzo(a,e)pyrene	0.05	0.17	0.03	0.09
Dibenzo(a,i)pyrene	0.18	0.60	0.09	0.30
Dibenzo(a,h)pyrene	0.06	0.20	0.03	0.10

Table S5. The values of exposed parameters used for the incremental lifetime cancer risk assessment (ILCR) [1]

Exposure parameter	Adult	Child	Unit
Body weight (BW)	61.5	15	kg
Exposure frequency (EF)	180	180	day $year^{-1}$
Exposure duration(ED)	24	6	year
Ingestion rate ($IR_{ingestion}$)	100	200	mg day^{-1}
Dermal exposure area (SA)	5700	2800	cm^2
Dermal adherence factor (AF)	0.07	0.2	mg cm^{-2}
Dermal adsorption fraction (ABS)	0.13	0.13	unitless
Inhalation rate ($IR_{inhalation}$)	20	10	m3 day-1
Average life span (AT) (70years×365days/year)	25550	25550	day
Particle emission factor (PEF)	1.36×10^9	1.36×10^9	m^3 kg^{-1}

Note: BW is body weight, AT is the average life span, EF is the exposure frequency, ED is the exposure duration, $IR_{inhalation}$ is the inhalation rate, $IR_{ingestion}$ is the dust intake rate, SA is the dermal surface exposure, AF is the dermal adherence factor, ABS is the dermal adsorption fraction, and PEF is the particle emission factor. CSF is defined as an upper-bound of the probability of a response per unit intake of a chemical over a lifetime.

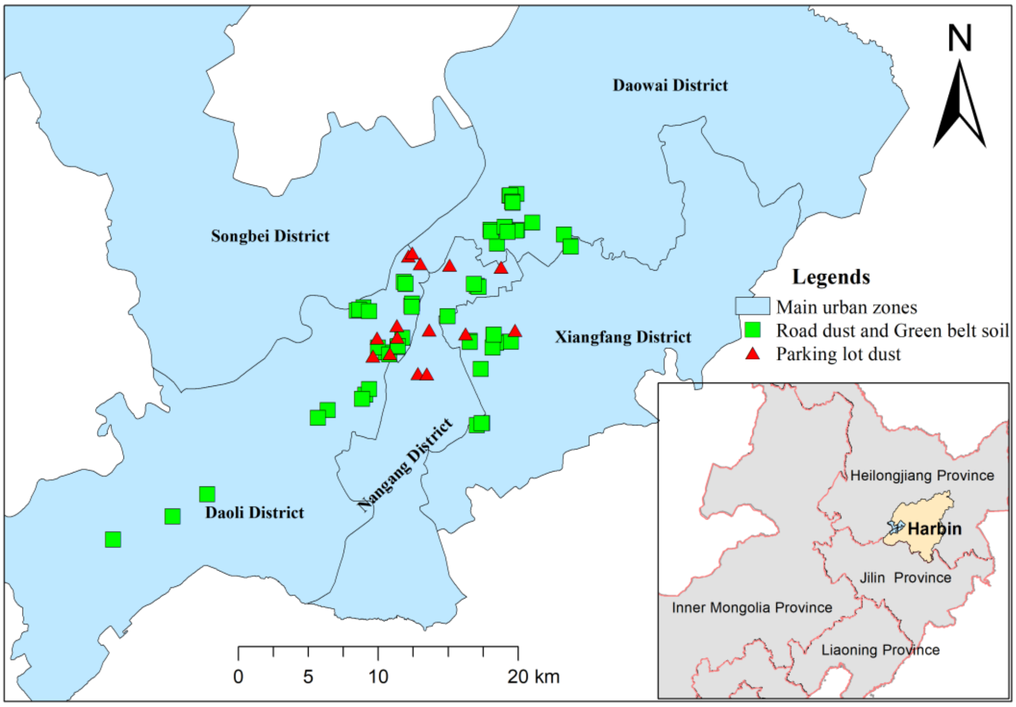

Figure S1. Sampling sites of road dust, green belt soil, and parking lot dust in Harbin, Northeast China

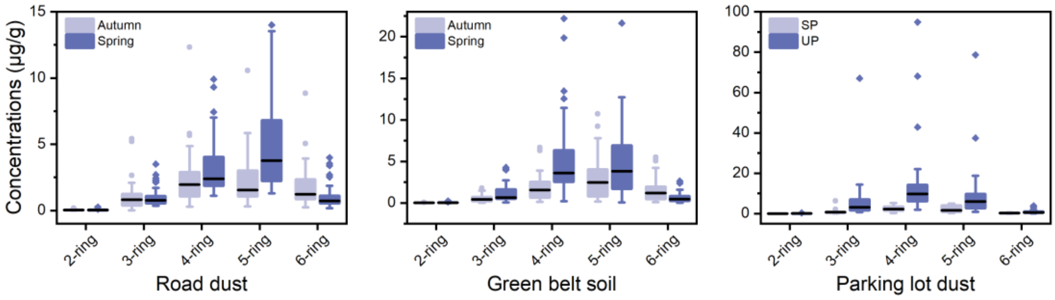

Figure S2. The concentrations of PAHs with a different number of aromatic rings in road dust, green belt soil, and parking lot dust.

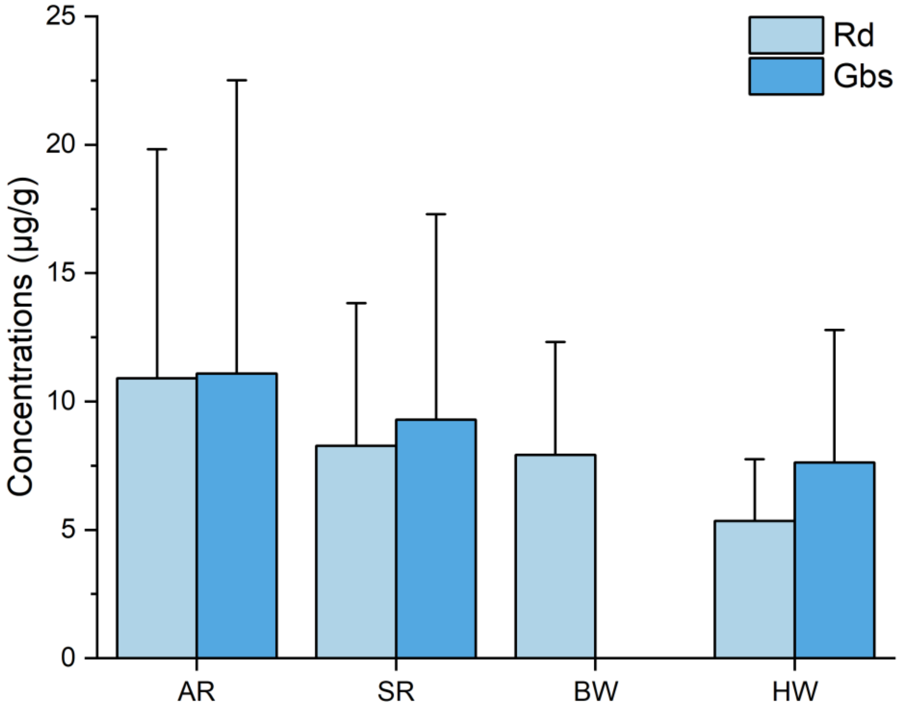

Figure S3. The concentrations of PAHs in AR, SR, BW, and HW during autumn and spring.

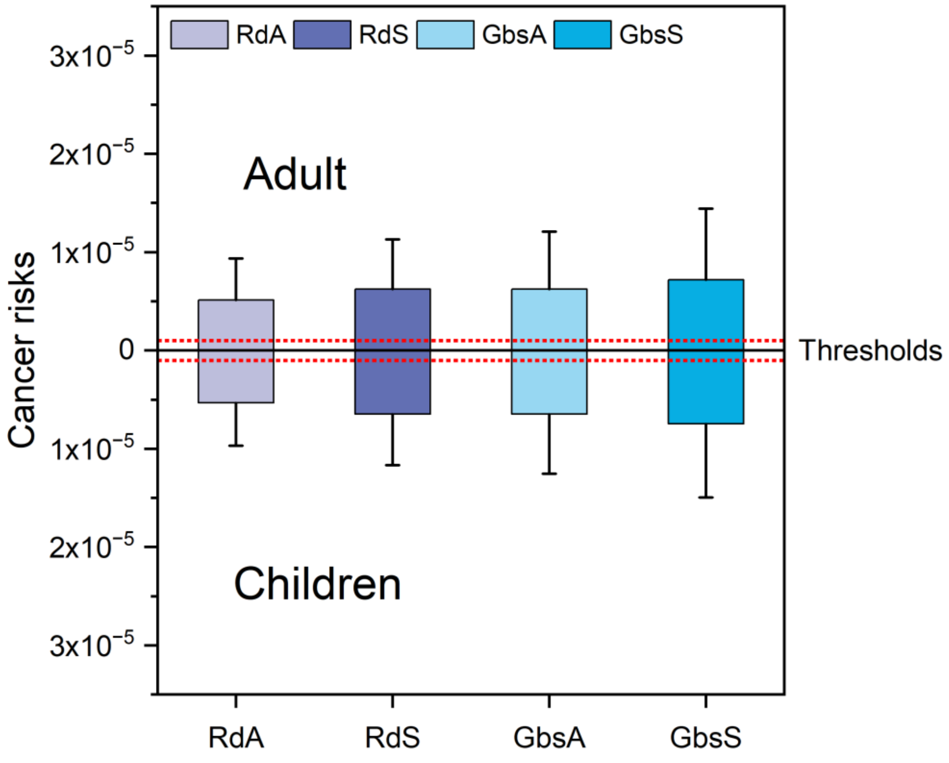

Figure S4. Seasonal variations of carcinogenic risk values.

RdA, road dust in autumn. RdS, road dust in spring. GbsA, green belt soil in autumn. GbsS, green belt soil in spring.

References

1. Wu, Z.; He, C.; Lyu, H.; Ma, X.; Dou, X.; Man, Q.; Ren, G.; Liu, Y.; Zhang, Y. Polycyclic aromatic hydrocarbons and polybrominated diphenyl ethers in urban road dust from Tianjin, China: pollution characteristics, sources and health risk assessment. Sustainable Cities and Society 2022, 81, https://doi.org/10.1016/j.scs.2022.103847.
